# Role of endoscopic biliary drainage in advanced hepatocellular carcinoma with jaundice

**DOI:** 10.1371/journal.pone.0187469

**Published:** 2017-11-02

**Authors:** Hyun Young Woo, Sung Yong Han, Jeong Heo, Dong Uk Kim, Dong Hoon Baek, So Yong Yoo, Chang Won Kim, Suk Kim, Geun Am Song, Mong Cho, Dae Hwan Kang

**Affiliations:** 1 Department of Internal Medicine, College of Medicine and Medical Research Institute, Pusan National University, Pusan National University Hospital, Busan, Republic of Korea; 2 Biomedical Research Institute, Pusan National University Hospital, Busan, Republic of Korea; 3 BIO-IT Foundry Technology Institute, Pusan National University, Busan, Republic of Korea; 4 Research Institute for Convergence of Biomedical Science and Technology, Pusan National University Yangsan Hospital, Yangsan, Republic of Korea; 5 Department of Radiology, College of Medicine and Medical Research Institute, Pusan National University, Pusan National University Hospital, Busan, Republic of Korea; Chang Gung Memorial Hospital Kaohsiung Branch, TAIWAN

## Abstract

**Background:**

Patients with advanced hepatocellular carcinoma (HCC) with jaundice have an extremely poor prognosis. Although biliary drainage can resolve obstructive jaundice, signs of obstruction may not be evident. This study evaluated the role of endoscopic biliary drainage in patients with advanced HCC and obstructive jaundice.

**Methods:**

From 2010 to 2015, 74 patients underwent endoscopic biliary drainage for obstructive jaundice due to advanced HCC. Jaundice resolution was defined as complete response and total bilirubin concentration below 3 mg/dl.

**Results:**

The technical success rate in the 74 patients was 92.1% (70/76). Of the 70 patients who underwent successful biliary drainage, 48 (68.6%) and 22 (31.4%) were Child-Pugh classes B and C, respectively, and 10 (14.3%) and 60 (85.7%) were BCLC stages B and C, respectively. Intrahepatic bile duct (IHD) dilatation was observed in 35 patients (50%). After drainage, the complete response rate was 35.7% (25/70). The mean time to resolution was 17.4 ±8.5 days. However, jaundice was re-aggravated in 74.3% (15/25) after a mean 103.5 ±96.4 days. Multivariate analysis showed that the absence of ascites, presence of IHD dilatation, normal range of prothrombin time, and lower MELD score were significantly associated with complete response. The overall survival rate was 15.7% (11/70) and the median survival time is 28 days (95% confidence interval 2.6–563 days). Complete response and HCC treatment after drainage were significantly associated with survival.

**Conclusion:**

Effective endoscopic biliary drainage is an important palliative treatment in patients with advanced HCC and obstructive jaundice, especially those with IHD dilatation and preserved liver function, as determined by ascites, prothrombin time, and MELD score.

## Introduction

Jaundice is encountered in 5–44% of patients with hepatocellular carcinoma (HCC) at the time of initial diagnosis and frequently occurs during the later stages of disease. Jaundice, which is associated with poor patient prognosis, is usually caused by diffuse tumor infiltration into the liver parenchyma, hilar invasion, and/or progressive terminal liver failure resulting from advanced underlying cirrhosis [1–8]. Obstructive jaundice, however, is uncommon, with only 0.5–13% of patients with HCC displaying definite obstructive jaundice. Obstructive jaundice may be caused by direct tumor invasion of the biliary tract, tumor casts, hemobilia, or extrinsic compression by a tumor or metastatic lymph nodes [1, 9].

Palliation of obstruction in patients with obstructive jaundice can improve quality of life and extend survival. Therefore, the cause of jaundice can affect patient prognosis [4]. However, signs of obstruction are not evident in many patients with obstructive jaundice, due to cirrhosis or diffuse tumor infiltration.

Although several studies have assessed the effects of endoscopic or percutaneous palliation of obstructive jaundice in small numbers of patients with HCC [1–8], few reports have assessed the effectiveness and durability of direct endoscopic biliary drainage without nasobiliary drainage in HCC patients with obstructive jaundice. This study therefore evaluated whether endoscopic biliary drainage could improve survival and offer the chance of subsequent HCC treatment in patients with HCC and obstructive jaundice, even in the absence of evident signs of obstruction. We also analyzed clinical and laboratory factors predicting effective endoscopic biliary drainage in these patients.

## Materials and methods

### Patients

This retrospective study evaluated patients who underwent biliary drainage via endoscopic retrograde cholangiopancreatography (ERCP) due to jaundice secondary to HCC invasion between January 2010 and February 2016. This study was in conformity with the ethical standards laid down in the 1964 Declaration of Helsinki and its later amendments and was approved by Institutional Review Board of Pusan National University Hospital (E-2016097). All patients gave their written informed consent before the treatment. HCC was diagnosed by histologic or non-invasive criteria, as defined by American Association for the Study of Liver Diseases guidelines [10]. Indication for ERCP for biliary drainage was serum total bilirubin concentration over 3 mg/dl and either the presence of definite intrahepatic duct (IHD) dilatation or a finding of bile duct invasion if IHD dilatation was not evident as determined by computed tomography or magnetic resonance imaging. IHD dilatation was defined as evident when an axial scan on computed tomography or magnetic resonance imaging showed that the lumen of the intrahepatic bile duct was dilated more than 2 mm, continuously from the proximal to the distal portion of the hepatic parenchyma [11]. All patients had symptoms of obstructive jaundice, such as dark brown or reddish urine, an itching sensation and pale stool. None of these patients had any viral or toxic cause of jaundice. Intrahepatic biliary obstruction was classified by mechanism, with type I defined as due to intraluminal obstruction, type II to hemobilia, and type III obstruction to extraluminal obstruction.

### Procedures and outcome measures

Initial evaluation included confirmation of the cause of underlying liver disease, liver function tests, determination of Child-Pugh score, measurement of serum α-fetoprotein (AFP) concentration, calculation of model for end-stage liver disease (MELD) score, and HCC staging including vascular invasion and nodal and distant metastases. Because the numerical value of prothrombin time fluctuated when jaundice appeared, but the degree of fluctuation was trivial, the worst prothrombin time before biliary drainage was used in the analysis. All ERCP procedures were performed within 30 days of presentation of obstructive jaundice by two endoscopists (DUK, DHB), using a duodenoscope (TJF-240, TJF-260, JF-240, Olympus Co., Ltd, Tokyo, Japan). Prophylactic antibiotics were administered to all patients who underwent ERCP and were maintained in patients suspected of infection.

Patients underwent standard biliary cannulation. Major endoscopic sphincterotomy was not performed due to high risk of bleeding tendency cause by impaired liver function. In most cases, endoscopic sphincterotomy was not performed or minor sphinterotomy was done. If standard biliary cannulation failed, needle-knife precut sphincterotomy was performed very cautiously with attention of bleeding. In case of bleeding during the procedure, endoscopic hemostasis was carried out appropriately. In most cases, a 7 Fr. plastic stent (Boston 135 Scientific, Natick, MA, USA) was inserted into the bile duct because none of these patients underwent major endoscopic sphincterotomy. Only two patients received uncovered self-expandable metal stents (Niti-S, Taewoong Med. Co., South Korea). Both plastic and metal stents were placed across the papilla, thereby making secondary procedures easier to perform in the event of stent occlusion. After the procedure, all patients were examined for procedure-related complications; occurrence of bleeding, post-ERCP pancreatitis, post-ERCP cholangitis and perforation by clinical symptoms, signs and measuring hemoglobin, C-reactive protein, serum amylase and lipase levels and by abdominal X-rays. Stent position after the procedure was confirmed one day later by abdominal X-rays. Clinical response after biliary drainage including the change of total bilirubin, albumin, prothrombin time, Child-Pugh score and MELD score was evaluated. Complete response was defined as a total bilirubin concentration <3 mg/dl within 30 days of ERCP. Clinical course after biliary drainage such as the durability of complete response, additional treatment for HCC, survival was also evaluated.

### Statistical analysis

Statistical analyses were performed using IBM SPSS statistical software, version 21.0 (SPSS, New York, NY, USA). Continuous variables were expressed as medians and ranges, and categorical variables as absolute numbers and relative frequencies. Qualitative and quantitative variables were compared using the χ2-test and the Mann—Whitney U-test, respectively. Logistic regression analysis was used to identify factors predicting successful biliary drainage. Survival probabilities were calculated by the Kaplan—Meier method and displayed graphically. Cox’s proportional hazards model was used to identify factors predicting survival. A two sided P value less than 0.05 was considered statistically significant.

## Results

### Technical outcomes of ERCP

**Fig 1:**
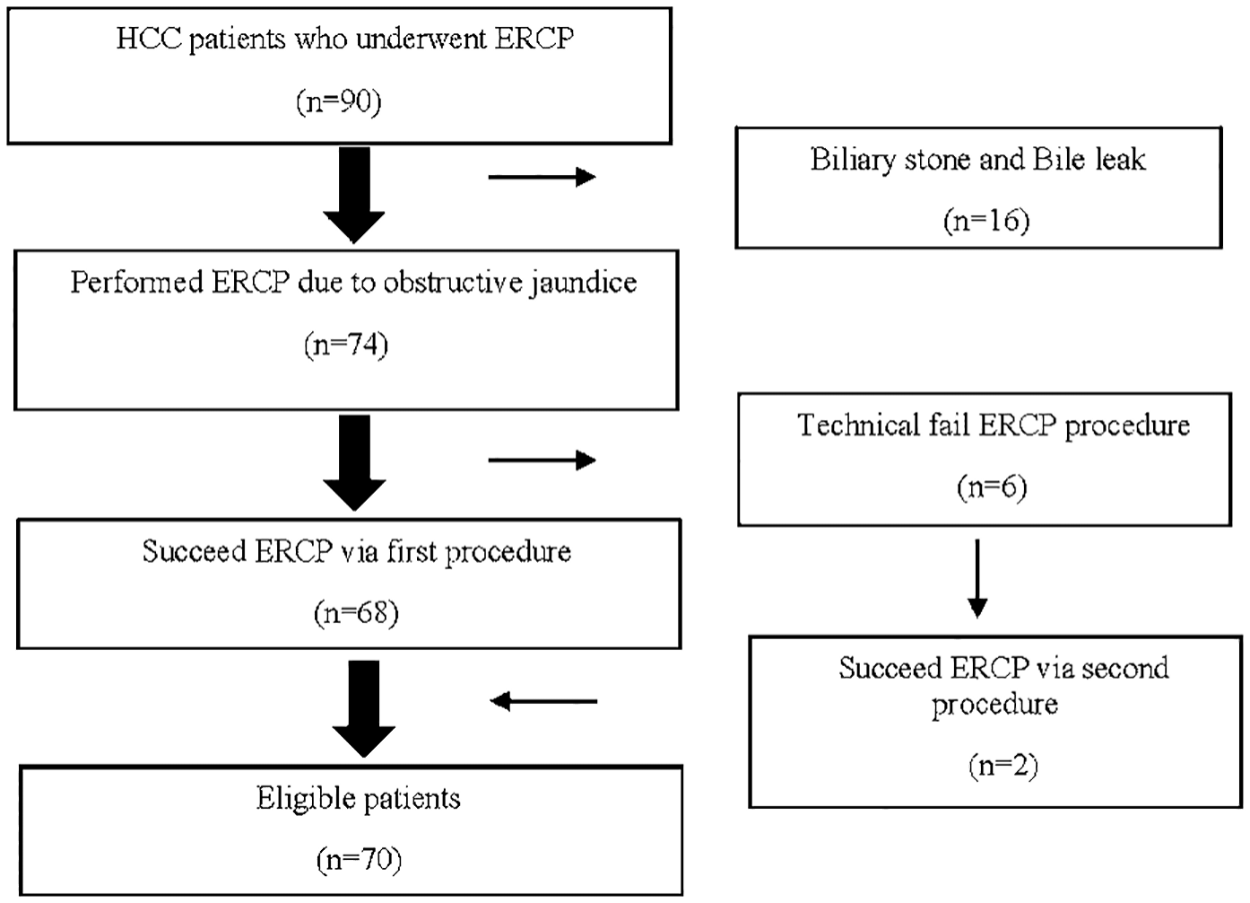
Study flow chart.

The study flow chart is shown in Fig 1. Initially, 74 HCC patients with naïve papilla underwent ERCP, with six experiencing technical failure. Two of these patients underwent repeat ERCP. Therefore, a total of 76 ERCP procedures were performed in 74 patients, with a technical success rate of 92.1% (70/76). Finally, 70 patients were enrolled the study. The four failures were caused by failure of bile duct cannulation in one patient, a failed approach to the duodenum due to deformity in two patients, and failed passage to IHD caused by an intraluminal mass in one patient. The rate of post-ERCP pancreatitis was 18.6% (13/70), with all of these patients having mild pancreatitis. Two patients (2.9%) with needle-knife precut sphincterotomy had bleeding complications, but neither required red blood cell transfusion and well managed with endoscopic hemostatsis. The first retrograde biliary tract infection rate after first biliary drainage was 1.4% (1/70). None of these patients experienced perforation.

### Patient characteristics

The baseline characteristics of the 70 included patients are summarized in Table 1. Mean ± SD patient age was 62±9.8 years and 61 patients (87.1%) were male. The major causes of HCC were chronic hepatitis B (62.8%) and chronic hepatitis C (22.9%). Of the 70 patients, 48 (68.6%) were classified as Child-Pugh class B and 22 (31.4%) as Child-Pugh class C and mean ± SD of Child-Pugh score is 8.8 ± 1.0. Thirty-six patients (51.4%) had ascites, and 35 (50%) had definite IHD dilatation. Prior to ERCP, these patients had a mean ± SD serum total bilirubin concentration of 9.6 ± 5.8 mg/dL, serum albumin level of 3.17 ± 0.95 g/dL, a mean ± SD prothrombin time (INR) of 1.36 ± 0.23, a mean ± SD aspartate transaminase concentration of 237.0 ± 199.0 IU/L, a mean ± SD alkaline phosphatase concentration of 340.2 ± 247.2 IU/L, and a mean ± SD MELD score of 18.4 ± 3.4. Sixty-three patients (90.0%) presented with cholangitis and eight (11.4%) with mycobacterial infection, including five with *Klebsiella pneumoniae*, one with *Pseudomonas aeruginosa*, and one each with *Escherichia coli* and *E*. *faecium*.

**Table 1:** Baseline characteristics.

Variables	n = 70 (%)
Male	61 (87.1)
Age, years	62.0±9.8
Etiology, HBV/HCV/unknown	44/16/10 (62.8/22.9/14.3)
Child-Pugh class, A/B/C	0/48/22 (0/68.6/31.4)
BCLC stage, A/B/C	0/10/60 (0/14.3/85.7)
Okuda, I/II/III	1/35/34 (1.4/50/48.6)
Tumor volume, >50%	37 (52.9)
Portal vein tumor thrombosis	47 (67.1)
Metastasis	17 (24.3)
Ascites	36 (51.4)
Encephalopathy	2 (2.9)
Prior HCC treatment history	52 (74.3)
Intrahepatic bile duct dilatation	35 (50)
Location, Total/Right/Left/Rt. segment	20/20/15/15 (28.6/28.6/21.4/21.4)
Obstruction mechanism, I/II/III^a^	51/12/7 (72.9/17.1/10)
Laboratory parameters	
White blood cell count, /uL	7542±3734
Aspartate transaminase, IU/L	237.0±199.0
Total bilirubin, mg/dL	9.58±5.80
Alkaline phosphatase, IU/L	340.2±247.2
Creatinine, mg/dL	0.87±0.37
Prothrombin time (INR)	1.36±0.23
C-reactive protein, mg/dL	4.0±3.1
Alpha-fetoprotein, IU/L	14838±29131
MELD score	18.4±3.4

HBV, hepatitis B virus; HCV hepatitis C virus; BCLC, BCLC, Barcelona Clinic Liver Cancer; HCC, hepatocellular carcinoma; MELD, Model For End-Stage Liver Disease.

^a^ Obstruction mechanism type I: bile duct invasion, type II: hemobilia, type III: extraluminal compression

### Clinical outcome of ERCP

The mean time between jaundice presentation and biliary drainage was 6.1 days (range 0–29 days). After first biliary drainage, nine patients (12.9%) underwent an additional ERCP due to clinical deterioration, with three of these showing clinical improvement after 2^nd^ stage of biliary drainage. Of the six patients who did not show clinical response, two underwent a third ERCP, but neither showed clinical improvement. After biliary drainage, 35 (50%) of the 70 patients showed reduction in total bilirubin and 25 (35.7%) showed complete responses in total bilirubin. Mean time to complete response after ERCP was 17.4 ±8.5 days, with mean total bilirubin concentration at the time of complete response being 1.9±0.6 mg/dL. In addition to bilirubin, liver function including Child-Pugh score and MELD score was significantly improved in these 25 patients with complete response (*P*<0.01). Meanwhile, albumin and prothrombin time was not improved significantly. Of the 25 patients who showed complete responses to ERCP, ten underwent further HCC treatment, including seven who received transarterial chemoembolization and three who were treated with sorafenib. The remaining 15 patients who showed complete responses to ERCP did not receive additional HCC treatments; these included five patients in poor general condition, five with re-aggravation during consideration of HCC treatment; two who died of infection (one each of pneumonia and colitis); two who were lost to follow-up; and one who refused further HCC treatment. Of the 25 patients who showed complete response to ERCP, 20 (80%) showed recurrence of jaundice 103.5 ±96.4 days later. Aggravation of jaundice was due to tumor progression in 15 patients, including eight who received further HCC treatment, and to plastic stent malfunction in five. The causes of plastic stent malfunction included blood clot, tumor thrombus or sludge; of these five patients, one received additional HCC treatment. Median time to re-aggravation was shorter in patients with plastic stent malfunction (52.2 days; range, 23–107 days) than in those with tumor progression (127.5 days; range, 9–367 days). Of these 20 patients who experienced recurrence of jaundice, 13 (65%) underwent additional ERCP, with 6 of these 13 (61.5%) showing complete response. The other seven patients were not in suitable condition for ERCP or refused further treatment. The infection rate after repeated ERCP due to clinical failure or re-aggravation was 12.5% (3/24).

#### Clinical factors associated with complete response after biliary drainage

Univariate analysis showed that factors significantly associated with complete response after biliary drainage were smaller tumor volume, earlier Okuda stage, lower Child-Pugh class, lower total bilirubin concentration, lower alkaline phosphatase concentration, normal range of prothrombin time, lower MELD score, absence of ascites, presence of IHD dilatation and type II or III obstruction mechanism. Multivariate analysis showed that absence of ascites, presence of IHD dilatation, normal range of prothrombin time, and lower MELD score were significantly associated with complete response (Table 2).

**Table 2:** Clinical factors associated with complete response after biliary drainage.

Variable	Favorable response, n = 25 (%)	Without favorable response, n = 45 (%)	P value^b^	P value^c^
Age, years	64.1±9.3	60.8±10.0	0.180	-
Male	21 (84)	40 (88.9)	0.560	-
Etiology, HBV/HCV/unknown	16/3/6 (64/12/24)	28/13/4 (62.2/28.8/8.8)	0.467	-
Tumor volume, >50%	9 (36)	28 (62.2)	0.038	0.181
Child-Pugh, B/C	22/3 (88/12)	26/19 (57.7/42.3)	0.014	0.632
BCLC stage, B/C	6/19 (24/76)	4/41 (8.8/91.2)	0.095	-
Okuda, I+II/III	18/7 (72/28)	17/28 (37.7/62.3)	0.008	0.592
Portal vein tumor thrombosis	15 (60)	32 (71.1)	0.345	-
Metastasis	4 (16)	13 (28.9)	0.234	-
Ascites	8 (32)	28 (62.2)	0.018	0.049
Prior HCC treatment history	19 (76)	33 (73.3)	0.807	-
Intrahepatic bile duct dilatation	19 (76)	16 (35.6)	0.002	0.002
Location, Total/Right/Left/Right. Segment	3/6/10/6 (12/24/40/24)	17/14/5/9 (37.8/31.1/11.1/20)	0.340	-
Obstruction mechanism, I/II/III^a^	14/8/3 (56/32/12)	37/4/4 (82.2/8.9/8.9)	0.039	0.134
Jaundice to ERCP days	4.4±4.2	7.1±7.3	0.092	-
White blood cell count, /uL	7158.8±2952.4	7754.9±4120.2	0.526	-
Aspartate transaminase, IU/L	182.8±213.8	267.1±186.1	0.102	-
Total bilirubin, mg/dL	7.64±3.79	10.65±6.45	0.046	0.051
Alkaline phosphatase, IU/L	424.0±329.7	293.6±174.0	0.033	0.296
Creatinine, mg/dL	0.81±0.36	0.90±0.37	0.335	-
Prothrombin time (INR)	1.22±0.10	1.43±0.25	0.001	0.029
C-reactive protein, mg/dL	4.38±3.55	3.73±2.76	0.392	-
Alpha-fetoprotein, IU/L	13234±26519	15793±30853	0.731	-
MELD score	16.44±2.18	19.51±3.45	0.001	0.034

HBV, hepatitis B virus; HCV hepatitis C virus; BCLC, BCLC, Barcelona Clinic Liver Cancer; HCC, hepatocellular carcinoma; MELD, Model For End-Stage Liver Disease; ERCP, endoscopic retrograde cholangiopancreatography

^a^ Obstruction mechanism type I: bile duct invasion, type II: hemobilia, type III: extraluminal compression

^b^ P-value by univariate analysis

^c^ P-value by multivariate analysis

Factors predicting complete response after biliary drainage were analyzed in subgroups of patients with and without IHD dilatation (Table 3). Of the 35 patients without IHD dilatation, six (17.1%) showed complete response to ERCP, with lower baseline aspartate aminotransferase (AST) concentration being significantly associated with complete response (*P* = 0.038). Of the 35 patients with IHD dilatation, 19 (45.7%) showed complete response. Multivariate analysis showed that factors significantly associated with higher complete response rate included type II or III obstruction (*P* = 0.001), lower baseline total bilirubin concentration (*P* = 0.011) and normal range of prothrombin time (*P* = 0.001).

**Table 3:** Subgroup analysis of clinical factors associated with complete response after biliary drainage according to IHD dilatation status.

Variable	Without IHD dilatation		P value	With IHD dilatation		P value
	Favorable response, n = 6 (%)	Without favorable response, n = 29 (%)		Favorable response, n = 19 (%)	Without favorable response, n = 16 (%)	
Age, years	59.3±9.5	61.0±9.4	0.701	65.6±8.9	60.6±11.4	0.150
Male	5 (83.3)	27 (93.1)	0.436	16 (84.2)	13 (81.3)	0.823
Etiology, HBV/HCV/unknown	4/1/1 (66.7/16.7/16.7)	15/11/3 (51.7/37.9/10.3)	0.788	12/2/5 (63.1/10.5/26.3)	13/2/1 (81.2/12.5/5.3)	0.152
Tumor volume, >50%	4 (66.7)	21 (72.4)	0.777	5 (26.3)	7 (43.8)	0.293
Child-Pugh, B/C	5/1 (83.3/16.7)	17/12 (58.6/41.4)	0.254	17/2 (89.4/10.6)	9/7 (56.2/43.8)	0.025^b^
BCLC stage, B/C	1/5 (16.7/83.3)	1/28 (3.4/96.6)	0.204	5/14 (26.3/73.7)	3/13 (18.7/81.3)	0.608
Okuda, I+II/III	4/2 (66.7/33.3)	9/20 (31/69)	0.100	14/5 (73.6/26.4)	8/8 (50/50)	0.157
Portal vein tumor thrombosis	3 (50)	21 (72.4)	0.282	12 (63.2)	11 (68.8)	0.738
Metastasis	0 (0)	9 (31.0)	0.113	4 (21.1)	4 (25.0)	0.789
Ascites	1 (16.7)	17 (58.6)	0.061	7 (36.8)	11 (68.8)	0.063
Prior HCC treatment history	6 (100)	20 (69.0)	0.113	13 (68.4)	13 (81.3)	0.402
Location, Total/Right/Left/Right. segment	2/2/1/1 (33.3/33.3/16.7/16.7)	14/11/1/3 (48.2/37.9/3.4/10.3)	0.650	1/4/9/5 (5.3/21/47.4/26.3)	3/3/4/6 (18.8/18.8/25/37.5)	0.851
Obstruction mechanism, I/ II/ III^a^	6/0/0 (100/0/0)	22/4/3 (75.8/13.9/10.3)	0.226	8/8/3 (42.1/42.1/15.8)	15/1/0 (93.7/6.3/0)	0.001^c^
Jaundice to ERCP days	5.83±3.31	7.82±7.84	0.549	3.89±4.41	5.75±6.35	0.317
White blood cell count, /uL	8530±3763	7890±4087	0.726	6725±2619	7510±4302	0.512
Aspartate transaminase, IU/L	167.3±62.4	337.0±187.7	0.038^c^	187.8±244.4	140.5±96.2	0.473
Total bilirubin, mg/dL	6.75±3.50	9.06±4.87	0.279	7.91±3.92	13.5±8.01	0.011^c^
Alkaline phosphatase, IU/L	356.1±219.8	296.7±179.1	0.481	445.5±359.9	287.8±169.8	0.118
Creatinine, mg/dL	0.97±0.63	0.88±0.26	0.548	0.76±0.23	0.94±0.52	0.178
Prothrombin time (INR)	1.21±0.49	1.44±0.27	0.051	1.23±0.12	1.42±0.20	0.001^c^
C-reactive protein, mg/dL	4.46±2.95	3.77±2.90	0.601	4.36±3.79	3.65±2.59	0.534
Alpha-fetoprotein, IU/L	11264±21485	21828±36293	0.500	13856±28421	3722±5583	0.200
MELD score	16.8±2.6	18.7±2.9	0.155	16.3±2.1	20.4±3.8	0.000^b^

HBV, hepatitis B virus; HCV hepatitis C virus; BCLC, BCLC, Barcelona Clinic Liver Cancer; HCC, hepatocellular carcinoma; MELD, Model For End-Stage Liver Disease; ERCP, endoscopic retrograde cholangiopancreatography; IHD, intrahepatic bile duct dilatation

^a^ Obstruction mechanism type I: bile duct invasion, type II: hemobilia, type III: extraluminal compression

^b^ P <0.05 in univariate analysis

^c^ P< 0.05 in uni- & multivariate analysis

### Clinical factors associated with survival

**Fig 2:**
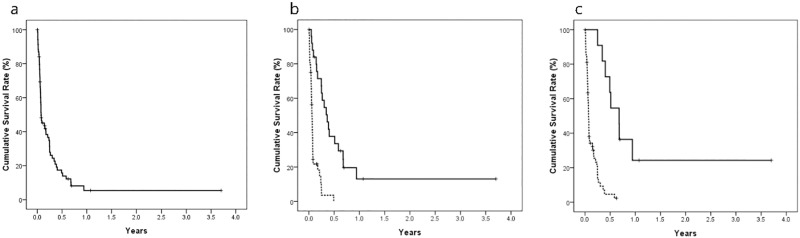
Cumulative survival rate of HCC patients who underwent biliary drainage. Cumulative survival rate in overall population (A) and according to complete response after biliary drainage (B) and HCC treatment after biliary drainage (C).

The overall survival rate in the study population was 15.7% (11/70), with a median survival time of 28 days (95% confidence interval [CI] 2.6–563 days) (Fig 2A). Univariate analysis showed that lower Child-Pugh class, earlier Okuda stage, lower BCLC tumor stage, smaller tumor volume, absence of ascites, presence of IHD dilatation, segmental tumor location, lower AST concentration, HCC treatment and complete response were significantly associated with survival (Table 4).

**Table 4:** Clinical factors associated with survival.

	Expired patients/All patients, median survival (95% CI)	P value^b^	P value^c^
Age, ≤65 />65 years	36/43, 28 days (21.9–34.0)/23/27, 33 days (0.0–78.0)	0.495	-
Sex, Male/Female	51/61, 27 days (20.6–33.3)/8/9, 33 days (0.0–81.5)	0.849	-
Cause, HBV/HCV/unknown	37/44, 29 days (2.5–55.4)/16/16, 17 days (9.1–24.8)/6/10, 130 days (33.4–226.5)	0.419	-
Tumor volume, ≤ 50%/>50%	27/33, 89 days (42.6–135.3)/32/37, 23 days (18.2–27.7)	0.002	0.154
Child-Pugh, B/C	38/48, 76 days (20.7–131.2)/21/22, 19 days (15.5–22.4)	0.000	0.091
BCLC stage, B/C	7/10, 95 days (60.8–129.1)/52/60, 27 days (22.8–31.1)	0.047	0.071
Okuda, I+II/III	30/35, 62 days (0–137.9)/29/35, 21 days (14.1–27.8)	0.036	0.446
Portal vein tumor thrombus, -/+	20/23, 55 days (0–138.2)/39/47, 27 days (23.1–30.8)	0.716	-
Metastasis, -/+	15/17, 55 days (14.8–95.1)/44/53, 27 days (24.1–29.8)	0.157	-
Ascites, -/+	31/36, 85 days (7.5–162.4)/28/34, 23 days (15.3–30.6)	0.028	0.876
History of previous HCC treatment, -/+	15/18, 63 days (0–143.6)/44/52, 28 days (23.4–32.5)	0.241	-
Intrahepatic bile duct dilatation, –/+	32/35, 21 days (13.0–29.0)/27/35, 90 days (40.9–139.0)	0.000	0.142
Location, Left/Right. Segment/Right/Total	12/15, 90 days (28.6–151.3)/10/15, 89 days (0–361.1)/19/20, 24 days (21.0–26.9)/18/20, 21 days (13.0–28.9)	0.032	0.815
Obstruction mechanism, I/ II/ III^a^	43/51, 24 days (21.2–32.7)/10/12, 95 days (1.0–188.9)/6/7 26 days (0.0–59.3)	0.748	
Aspartate transaminase, IU/L, ≤150/>150	24/30, 90 days (84.0–95.9)/35/40, 23 days (17.8–28.1)	0.002	0.803
Total bilirubin, mg/dL, ≤10/>10	41/47, 28 days (19.3–36.6)/18/23, 30 days (0–81.8)	0.929	-
Alkaline phosphatase, IU/L, ≤390/>390	43/50, 27 days (19.3–34.6)/16/20, 52 days (0–106.9)	0.206	-
C-reactive protein, ≤4/ >4	38/44, 29 days (20.5–37.4)/21/26, 27 days (0–68.3)	0.908	-
MELD score, ≤18/ >18	35/40, 52 days (12.1–91.8)/24/30, 26 days (19.8–32.1)	0.105	-
Complete response, –/+	39/45, 22 days (17.9–26.0)/20/25, 130 days (77.7–182.2)	0.000	0.002
HCC treatment, –/+	51/59, 25 days (21.3–28.6)/8/11, 244 days (170.6–317.3)	0.000	0.000

HBV, hepatitis B virus; HCV hepatitis C virus; BCLC, BCLC, Barcelona Clinic Liver Cancer; HCC, hepatocellular carcinoma; MELD, Model For End-Stage Liver Disease; ERCP, endoscopic retrograde cholangiopancreatography

^a^ Obstruction mechanism type I: bile duct invasion, type II: hemobilia, type III: extraluminal compression

^b^ P-value by univariate analysis

^c^ P-value by multivariate analysis

Multivariate analysis showed that complete response after biliary drainage and HCC treatment after biliary drainage were significantly associated with survival. Median survival was significantly longer in patients with than without complete response (130 days; 95% CI 77.7–182.2 days vs. 22 days; 95% CI 17.9–26.0 days; *P* = 0.002) (Fig 2B). Overall, 10 patients with and one without complete response received further HCC treatment. Median survival was significantly longer in patients who did than did not receive further HCC treatment [244 days; 95% CI 170–317 days vs. 25 days; 95% CI 21–28 days; *P*<0.001) (Fig 2C). Median survival was especially longer in patients who did than did not receive HCC treatment after complete response [244 days; 95% CI 149–338 days vs. 89 days; 95% CI 28–149 days; *P* <0.001).

## Discussion

The prognosis of advanced HCC patients with obstructive jaundice is poor, with many patients experiencing hepatic failure due to poor hepatic functional reserve [1]. Palliative biliary drainage would therefore benefit HCC patients with obstructive jaundice. In addition, palliative biliary drainage would increase the possibility of these patients receiving additional treatment for HCC and prolong their survival [6]. Effective drainage is important in the management of HCC accompanied by obstructive jaundice [1–8]. In these studies, IHD dilatation was an indicator of obstruction in most patients. Clinically, however, it may be difficult to determine the cause of jaundice. Signs of obstruction such as IHD dilatation may be masked by cirrhosis or diffuse tumor infiltration. Moreover, in many patients, jaundice may consist of both obstructive and non-obstructive jaundice. These findings indicate that IHD dilatation alone should not be an absolute indicator of biliary drainage. In this study, half of patients without IHD dilatation underwent biliary drainage, with some showing complete response. Therefore, the clinical outcomes of patients in this study may reflect the actual clinical course of patients with jaundice associated with advanced HCC who undergo biliary drainage.

Endoscopic retrograde biliary drainage (ERBD) and percutaneous transhepatic biliary drainage (PTBD) are the two main drainage procedures in HCC patients with obstructive jaundice. ERBD is considered first-line treatment [6], with PTBD being second-line treatment in patients who fail ERBD or those in whom ERBD is impossible to perform. A comparative study showed that survival was longer following ERBD than PTBD, due to the higher success rate of effective drainage and longer duration of stent patency in the ERBD group [3]. The reported success rates of ERBD in providing effective drainage were 54–72% [3, 7, 12, 13]. In our study, the technical success rate was 92.1% (70/76) and the clinical success rate was 35.7% (25/70). The lower complete response rate in this study than in other studies was likely due to the performance of biliary drainage in nearly half of patients without definite signs of obstructive jaundice.

Some patients might undergo these procedures during progression to hepatic failure rather than for obstructive jaundice. In 20 patients, the tumor occupied the entire liver. The complete response rate was lower in this group, with 12% of patients having a favorable response and 37.8% having a non-favorable response, *P* = 0.028) (S1 Table). The median survival time in this group was 21 days (range, 13–28.9 days), similar to that in patients without a complete response (22 days; range, 17.9–26 days). However, the median survival of the three patients in this group with complete response was 90 days, compared with 19 days in patients without a complete response, although the rate of complete response after biliary drainage in those patients was only 15% (3/20). The main problem was the difficulty of determining the cause of jaundice in this group, hepatic failure or obstructive jaundice. Therefore, biliary drainage of all patients in this group may be questionable due to its low efficacy and the risks associated with an invasive procedure. The results presented in this study suggest the need for a careful and individualized approach.

The complete response rate was significantly lower in the absence than in the presence of IHD dilatation (17.1% [6/35] vs. 54.3% [19/35]). Therefore, the presence of IHD dilatation was predictive of the resolution of jaundice after palliative drainage. Previous studies, however, showed that effective drainage was the only way to improve survival in these patients with jaundice. Thus, even if patients with jaundice do not show definite IHD dilatation, biliary drainage should be considered in those suspected of obstruction. We found that, in addition to IHD dilatation, the resolution of jaundice after biliary drainage in the overall population was significantly higher in patients without ascites, those with a normal range of prothrombin time, and patients with lower MELD scores. Subgroup analysis of patients with IHD dilatation showed that the resolution of jaundice after biliary drainage was significantly higher in patients with type II or III obstruction and those with lower baseline total bilirubin concentration. In patients without IHD dilatation, the resolution of jaundice was higher in patients with lower AST concentration. Almost all previous studies assessing biliary drainage in HCC patients with obstructive jaundice found that preserved liver function was an important predictor of response [1, 2, 4, 6, 14]. Preserved liver function in these patients indicates that jaundice was not caused by liver failure, although early liver failure may cause isolated jaundice. In these patients, other predictive factors, such as obstruction mechanism and AST level, baseline total bilirubin concentration would be helpful for differential diagnosis.

Previous studies of the effects of biliary drainage in HCC patients with obstructive jaundice have shown that median survival was about 1.3–1.5 months in patients with ineffective drainage and about 4.9–8.7 months in patients with effective drainage [2, 4, 6, 8]. Median survival was further prolonged, to about 8–13.4 months, in patients with effective drainage who received HCC treatment [14–16]. Similarly, in this study, survival time was considerably longer in patients with than without complete response (130 vs. 22 days) and in patients with complete response who did than did not receive subsequent treatment for HCC (244 vs. 89 days). Successful palliative drainage can therefore provide HCC patients with jaundice unexpected opportunities for extended survival, not only through successful drainage itself but the opportunity to receive additional HCC treatment. However, in comparing baseline characteristics, we found that tumor volume was significantly larger and history of previous HCC treatment greater in patients who did not receive additional HCC treatment (S2 Table). Therefore, further studies are necessary to determine whether HCC treatment after biliary drainage prolongs patient survival.

Previous studies have shown that, in addition to successful drainage subsequent treatment for HCC, liver function and HCC tumor stage were significant predictors of survival, although factors differed among the studies [2–4, 6, 8]. The present study found that liver function (Child-Pugh score, presence of ascites) and HCC size (tumor volume, HCC stage) were significant predictors of survival in univariate, but not in multivariate analysis. Most patients with obstructive jaundice had advanced stage HCC, as determined by tumor stage and liver function. More than 85% of patients presented with BLCL stage C, and with homogeneous Child-Pugh and MELD scores. This may explain why only successful drainage and the presence of subsequent treatment for HCC were found to be significant in multivariate analysis.

The only drainage methods used to treat patients in this study were ERBD and biliary stent insertion. None of these patients underwent PTBD or endoscopic nasobiliary drainage (ENBD). Other studies of biliary drainage in patients with HCC used a greater number of methods, including PTBD, ERBD and ENBD. ENBD is a temporary method and is associated with patient discomfort. Following resolution of jaundice by this method, patients require another procedure to maintain drainage. The PTBD method includes penetration of the liver parenchyma and maintenance of an external drainage bag, reducing patient quality of life. Moreover, because PTBD requires penetration of the skin, it can also cause bleeding and infection. Direct ERBD drainage may be more comfortable for patients over the long term but requires skilled operators for catheter passage. Moreover, the rate of complications, including bleeding, post-ERCP pancreatitis and perforation, may be higher with ERBD than with ENBD. In this study, rates of post-ERCP pancreatitis and bleeding were 18.6% and 2.9%, respectively. The rate of bleeding in this study might be favorable, as most patients in this study had advanced liver cirrhosis. The rate of post-ERCP pancreatitis is a little higher compared to previous report (about 2–20%) [17]. The reason of this higher rate might be because endoscopic sphincterotomy was not performed or minor sphinterotomy was done in most cases of this study. However, none of the patients in this study experienced severe complications that affected their prognosis. Another shortcoming of direct ERBD drainage is its limited durability due to obstruction by a blood clot or tumor thrombus. However, the median durability of ERBD in this study was 2–3 months, comparable with previous studies. Lastly, stent placement in malignant strictures is a risk factor for post-ERCP cholangitis. In this study, the retrograde biliary tract infection rate after first biliary drainage was 1.4% (1/70) and the infection rate after repeated ERCP due to clinical failure or re-aggravation was 12.5% (3/24). The rate of post-ERCP cholangitis was found to be ≤1%, but the infection rate in patients with malignant strictures has been reported to be as high as 18% [18, 19]. Therefore, the infection rate in this study was comparable to that in previous studies, although this rate was found to increase after repeated ERCP. Additionally, regarding the stent placement, all stents in this study were placed across the papilla, thereby making secondary procedures easier to perform in patients with stent occlusion. The effect of this stent position on retrograde biliary tract infection seems trivial, but further studies are necessary to clarify this issue.

This study had several limitations, including the relatively small number of patients and the lack of a predesigned protocol for biliary drainage. This study included patients both with and without IHD dilatation, who were not well-matched in baseline characteristics, including tumor stage and liver function. However, because biliary drainage is the only palliative care method for patients with advanced HCC and obstructive jaundice, and because successful drainage is the only method of improving survival, well-designed prospective randomized studies would be difficult to perform.

In conclusion, effective endoscopic biliary drainage is an effective palliative treatment in patients with HCC and obstructive jaundice. Distinguishing among causes of jaundice and performance of early endoscopic biliary drainage in patients with obstruction are important. The presence of IHD dilatation and preserved liver function, as determined by ascites, prothrombin time, and MELD score, are important predictors of the effectiveness of endoscopic biliary drainage in HCC patients with obstructive jaundice.

## Supporting information

S1 Table Complete response after biliary drainage according to HCC location. (DOCX)

S2 Table Baseline characteristics according to presence of subsequent HCC treatment. (DOCX)
