# Supplementary material for: Role of endoscopic biliary drainage in advanced hepatocellular carcinoma with jaundice
Source: PLoS One. 2017 Nov 2;12(11):e0187469. doi: 10.1371/journal.pone.0187469 (PMC5667855; doi:10.1371/journal.pone.0187469)
Supplement: S1 Table — (DOCX) [file pone.0187469.s001.docx]

**S1 Table. Complete response after biliary drainage according to HCC location**

| Variable | Favorable response, n=25 (%) | Without favorable response, n=45 (%) | P-value |
| --- | --- | --- | --- |
| Location, Total/Right+Left+Right. Segment | 3/22 (12/88) | 17/28 (37.8/62.2) | 0.028 |
