# Supplementary material for: Role of endoscopic biliary drainage in advanced hepatocellular carcinoma with jaundice
Source: PLoS One. 2017 Nov 2;12(11):e0187469. doi: 10.1371/journal.pone.0187469 (PMC5667855; doi:10.1371/journal.pone.0187469)
Supplement: S2 Table — (DOCX) [file pone.0187469.s002.docx]

**S2 Table. Baseline characteristics according to presence of subsequent HCC treatment**

| Variable | Complete response | | P-value |
| --- | --- | --- | --- |
|  | With HCC treatment n=10 (%) | Without HCC treatment, n=15 (%) |  |
| Age, years | 61.5 (49-77) | 66 (44-78) | 0.567 |
| Male | 9 (90) | 12 (80) | 0.626 |
| Etiology, HBV/HCV/unknown | 7/2/1 (70/20/10) | 9/1/5 (60/6.7/33.3) | 0.310 |
| Tumor volume, >50% | 9/1 (90/10) | 7/8 (46.7/53.3) | 0.027 |
| Child-Pugh, B/C | 10/0 (100/0) | 12/3 (80/20) | 0.132 |
| BCLC stage, B/C | 2/8 (20/80) | 4/11 (26.7/73.3) | 0.702 |
| Okuda, I+II/III | 8/2 (80/20) | 10/5 (66.7/33.3) | 0.467 |
| Portal vein tumor thrombosis | 6 (60) | 9 (60) | 1.000 |
| Metastasis | 0 (0) | 4 (26.7) | 0.075 |
| Ascites | 2 (20) | 6 (40) | 0.294 |
| Prior HCC treatment history | 5 (50) | 14 (93.3) | 0.013 |
| IHD dilatataion | 9 (90) | 10 (66.7) | 0.181 |
| Location, Total/Right/Left/Right. segment | 1/1/4/4 (10/10/40/40) | 2/5/6/2 (13.3/33.3/40/13.3) | 0.363 |
| Obstruction mechanism, I/ II/ III^a^ | 6/3/1 (60/30/10) | 8/5/2 (53.3/33.3/13.3) | 0.940 |
| Jaundice to ERCP days | 4 (1-9) | 2 (0-15) | 0.807 |
| White blood cell count, /uL | 5065 (3820-10010) | 8730 (3070-13200) | 0.091 |
| Aspartate transaminase, IU/L | 104 (45-193) | 141 (43-1077) | 0.080 |
| Total bilirubin, mg/dL | 7.59 (3.05-13.01) | 5.08 (3.60-17.15) | 0.338 |
| Alkaline phosphatase, IU/L | 252.5 (82-683) | 318 (134-1635) | 0.367 |
| Creatinine, mg/dL | 0.75 (0.42-0.99) | 0.72 (0.38-2.11) | 0.683 |
| Prothrombin time (INR) | 1.18 (1.03-1.40) | 1.25 (1.15-1.40) | 0.091 |
| C-reactive protein, mg/dL | 3.54 (0.40-14.33) | 3.87 (0.76-11.57) | 0.531 |
| Alpha-fetoprotein, IU/L | 393 (2.1-27193) | 410 (1.38-1000000) | 0.723 |
| MELD score | 15 (11-19) | 17 (13-21) | 0.391 |

HBV, hepatitis B virus; HCV hepatitis C virus; BCLC, BCLC, Barcelona Clinic Liver Cancer; HCC, hepatocellular carcinoma; MELD, Model For End-Stage Liver Disease; ERCP, endoscopic retrograde cholangiopancreatography; IHD, intrahepatic bile duct dilatation

^a^ Obstruction mechanism type I : bile duct invasion, type II : hemobilia, type III : extraluminal compression
